# Oligodendrocyte lineage cells and depression

**DOI:** 10.1038/s41380-020-00930-0

**Published:** 2020-11-03

**Authors:** Butian Zhou, Zhongqun Zhu, Bruce R. Ransom, Xiaoping Tong

**Affiliations:** 1 Center for Brain Science, Shanghai Children’s Medical Center; Department of Anatomy and Physiology, Shanghai Jiao Tong University School of Medicine, Shanghai, China; 2 Department of Cardiothoracic Surgery, Center for Brain Science, Shanghai Children’s Medical Center, Shanghai Jiao Tong University School of Medicine, Shanghai, China; 3 Neuroscience Department, City University of Hong Kong, Hong Kong, China

**Keywords:** Depression, Cell biology, Neuroscience

## Abstract

Depression is a common mental illness, affecting more than 300 million people worldwide. Decades of investigation have yielded symptomatic therapies for this disabling condition but have not led to a consensus about its pathogenesis. There are data to support several different theories of causation, including the monoamine hypothesis, hypothalamic–pituitary–adrenal axis changes, inflammation and immune system alterations, abnormalities of neurogenesis and a conducive environmental milieu. Research in these areas and others has greatly advanced the current understanding of depression; however, there are other, less widely known theories of pathogenesis. Oligodendrocyte lineage cells, including oligodendrocyte progenitor cells and mature oligodendrocytes, have numerous important functions, which include forming myelin sheaths that enwrap central nervous system axons, supporting axons metabolically, and mediating certain forms of neuroplasticity. These specialized glial cells have been implicated in psychiatric disorders such as depression. In this review, we summarize recent findings that shed light on how oligodendrocyte lineage cells might participate in the pathogenesis of depression, and we discuss new approaches for targeting these cells as a novel strategy to treat depression.

## Introduction

Depression is a common mental disorder and the leading cause of ill health and disability worldwide, affecting with more than 300 million people. The number of depressed patients increased by over 18% from 2005 to 2015 [1]. In 2008, the World Health Organization (WHO) ranked major depressive disorder (MDD)as the third most common contributor to the global burden of disease and predicted that it would be ranked first by 2030 [2]. Depression is a serious mental illness involving persistent sadness or loss of interest or pleasure, accompanied by several of the following symptoms: disturbed sleep or appetite, feelings of guilt or low self-worth, tiredness, hopelessness, poor concentration, difficulty in making decisions, agitation or physical restlessness, and slowed speech or movement. Severe, depression can lead to suicide. Major depressive disorder (MDD) in adolescents between 10 and 19 years of age is associated with a high risk of lifelong disability and is a major factor leading to suicide [3], the second or third leading cause of death in this age group [4]. Nearly 800,000 people die of suicide caused by depression every year [5]. Depression often diminishes school and work performance, and interferes with healthy family interactions [3]. The global economic cost of depression in terms of lost productivity and required medical treatments is estimated to be $230 billion annually [6]. The greatest toll of depression is the human suffering experienced by patients and their families.

Although great advances have been made in the medical treatment of depression symptoms, there are no treatments that can be considered curative. There are many reasons for this. First, the clinical manifestations of depression are complex and variable, and causation may be multifactorial. Therefore, successful antidepressant drug assignment is made challenging by differences in clinical response between various forms of acute and chronic depression. Second, there are no known biomarkers that reliably predict whether specific therapeutic agents will be highly efficacious in a given patient. To date, the best animal models remain distant approximations of human depression [7]. Although most experimental hypotheses on the pathophysiology of depression have focused exclusively on neurons, including the monoamine, neuroplasticity and neurogenesis hypotheses as well as the hypothesis of hypothalamic–pituitary–adrenal (HPA) axis changes [8], the development of new drugs to treat depression has been stalled for decades, largely because the mechanisms of depression are still incompletely understood. More recently, however, studies have begun to suggest that glial cells might participate in the pathogenesis of depression. Astrocytes, for example, have been suggested to participate in depression via several distinct mechanisms, including mediating neuroinflammation and metabolic dysfunction and causing potassium channel-driven neuronal bursts [9–12]. Oligodendrocytes (OLs) have also been implicated in the pathogenesis of depression; in the following paragraph, we will review the evidence for this link.

Glial cells are a diverse group of nervous system cells. In the central nervous system (CNS), they are conventionally divided into macroglia, primarily astrocytes and OLs, and microglia. The glial cells in the brain outnumber the neurons and constitute roughly half the volume of the CNS of mammals [13]. OLs are mature, terminally differentiated cells that form myelin sheaths around axons. They are found predominantly, but not exclusively, in CNS white matter, the portion of the brain and spinal cord that contains exclusively axons. OLs produce myelin sheaths that allow ‘saltatory’ action potential propagation, which greatly increases conduction velocity, enhancing the speed and efficiency of communication between CNS neurons [14]. In the CNS, oligodendrocyte progenitor cells (OPCs) mainly evolve into OLs. Numerous OPCs (also called NG2 glia) are present in mature brains, constituting ~5% of total neural cells, and retain the capacity for self-proliferation throughout life [15]. OL lineage cells therefore include both OPCs and OLs. The expanding repertoire of OL lineage cell functions includes trophic support of ensheathed axons, formation of myelin, ionic homeostasis, synaptic transmission, brain energy metabolism and learning and memory [13, 16–19]. All these explicitly identified functions have led to theories and studies that focus closely on exploring relationship between OL lineage cells and depression. In this review, to provide a better understanding of how OL lineage cells participate in the pathogenesis of depression, we will briefly summarize the origin and developmental characteristics of these cells in the brain.

## The development of oligodendrocyte lineage cells

In the mammalian CNS, OL lineage cells include mature OLs and OPCs. OPCs are also called NG2 glia because they are identified by the expression of NG2-proteoglycan (also known as CSPG4) on the cell surface. They constitute a fourth large glial cell population in the CNS, distinct from mature OLs, astrocytes, and microglia [20]. Pericytes also express the NG2-proteoglycan; therefore, OL lineage cells that express this marker are best referred to as “NG2 glia” for the sake of precision [21, 22]. Recent studies have shown that the origin and differentiation of OPCs are different during mouse and human embryonic development. There are two waves of OPC generation in the human brain. Early OPCs originate from 9 gestational weeks (gw) in the ganglionic eminence and then spread to the cortex during the next few weeks [23–25]. Late OPCs appear in modest numbers at ~15 gw in both ganglionic eminences and cortical ventricular/subventricular zone (VZ/SVZ) [23–25]. The first myelin sheaths can be identified by myelin basic protein (MBP) expression at 18 gw in the thalamus and ~21 gw in the internal capsule [23–25]. At this stage, OPCs express platelet-derived growth factor receptor α (PDGFRα), the transcription factor SOX10, and NG2-proteoglycan and can be identified by the A2B5 antibody as well [26–28]. However, in studies using histological and fate mapping in Cre-loxP transgenic mice, OPCs arising from three different sources in continuous waves during early development have been found in the mouse forebrain. The first wave of OPCs is derived from the medial ganglionic eminence and anterior entopeduncular area in the ventral forebrain at approximately E11.5 (embryonic day 11.5). The second wave of OPCs arises from the lateral and/or caudal ganglionic eminences (LGE and CGE) at E15. The third wave arises from Emx1‐positive cells within the postnatal cortex [29].

Through in vitro and in vivo fate mapping, studies revealed that OPCs mainly proliferate and differentiate into mature OLs throughout their lifetime to form the myelin sheath. A number of transcription factors, such as Olig1, Olig2, NKX2.2, SOX9, SOX10, zinc finger protein 24 (Zfp24), and MYF, have been found to perform different functions during this process [30]. Olig2 is expressed in OPCs throughout development, postnatal differentiation and in mature OLs (at lower levels), and it can be combined with evolutionarily conserved U2 enhancer in the distal 5′-flank of the SOX10 gene and activates its transcription. SOX10 is expressed throughout the development, differentiation and maturation of OL lineage cells and has also been recognized as a major determinant in the final differentiation of OLs [28, 30, 31].

After OPCs generate OLs, the differentiated OLs can be divided into two stages: premyelinating and myelinated OLs. At the postmitotic stage of the OL lineage, myelin gene regulatory factor (MRF), as an OL-specific target of SOX10, plays a critical role in the progeny of myelinated OLs [32, 33]. The premyelinating OLs repress the expression of NG2, A2B5 and PDGFRα and begin to express antigen O4. At the same time, premyelinating OLs start to express galactocerebroside, MBP, proteolipid protein (PLP), myelin-associated glycoprotein (MAG) and/or myelin OL glycoprotein (MOG) to form the myelin sheath around axons and finally differentiate into mature OLs [26].

Although OPCs mainly proliferate and differentiate into mature OLs, they can also generate astrocytes and neurons depending on the developmental stage and brain region. In a study using transgenic mice that are NG2creBAC or Cre reporter lacZ/EGFP (enhanced green fluorescent protein) (Z/EG) to trace the in vivo fate of OPCs, the Cre reporter EGFP could be detected in a subpopulation of protoplasmic astrocytes in the gray matter of the ventrolateral forebrain but not in fibrous astrocytes of white matter [34]. In addition, in PDGFRα-CreER^T2^;RosaYFP double-transgenic adult mice, a small number of YFP+ neurons could be detected in the forebrain, especially in the piriform cortex, which is the main projection target of the olfactory bulb [35]. However, another study reported that early embryonic OPCs are restricted to the OL lineage and cannot generate neurons in the cerebellum, brain stem or olfactory bulb [36]. These contradictory observations necessitate further in-depth investigation in the field.

In the CNS, whether OL lineage cells are a heterogeneous or homogeneous cell population remains controversial as well. By using bulk RNA-seq analysis and single-cell transcriptomic analysis of mouse forebrain and spinal cord *Pdgfra*+ cells at embryonic and postnatal stages, a study showed that embryonic *Pdgfra*+ cells produce mainly OL progenitor cells during development, while the postnatal OPCs arising from different parts of the CNS are highly similar to one another in that they show similar transcriptional and electrophysiological properties, highlighting the homogeneity of OPCs in the brain [37–40]. However, other studies have strongly supported the idea that both OPCs and OLs possess heterogeneous characteristics. For instance, Spitzer et al., using single-cell electrophysiological recordings, found that a homogeneous population of OPCs becomes functionally heterogeneous both within and between brain regions and with age. The electrophysiological changes in OPCs are directly correlated with the differentiation potential of OPCs [41, 42]. In addition, Marques et al. recently reported that diverse subsets of mature OLs are enriched in specific regions of the adult brain by performing single-cell RNA sequencing on 5072 cells of the OL lineage obtained from ten regions of the mouse juvenile and adult CNS [43]. Among the intrafascicular OLs, they can also be divided into four types (type I to type IV), depending largely on the defined brain region, myelin sheath segments, ion channel and membrane receptor expression, which illustrates the dynamics of OL differentiation and heterogeneity in the CNS [44]. Typically, OL lineage cells rapidly react to various insults including brain stab injury [45, 46], spinal cord injury [47], and demyelination injury [48, 49], as well as a model of ALS [50], by changing their morphology, proliferation and/or differentiation rate. Nevertheless, much of what has been described as heterogeneity in OL lineage cells is based on phenotypic differences. Without evidence for functional differences between putative subgroups of OL lineage cells, distinguishing heterogeneity from plasticity and lineage state is difficult [51].

**Fig. 1:**
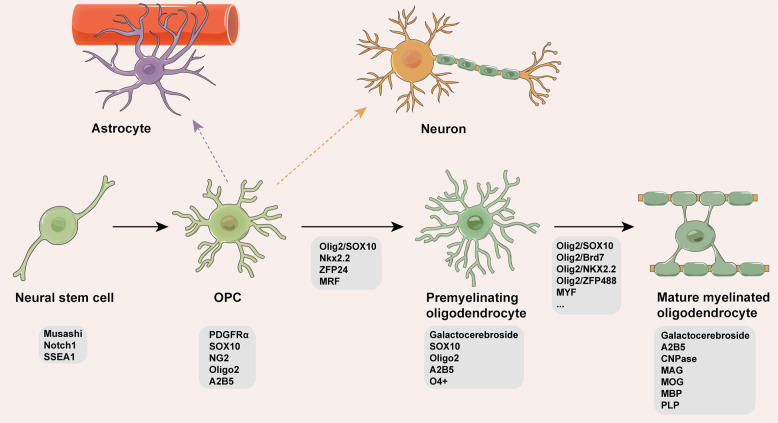
Differentiation and proliferation of oligodendrocyte lineage cells. Based on the expression of different special proteins, the origin and development of oligodendrocyte lineage cells can be subdivided into three stages: oligodendrocyte progenitor cells (OPCs), premyelinating oligodendrocytes and mature oligodendrocytes (OLs). Originating from neural stem cells, OL lineage cells begin to express PDGFRα, NG2 (OPCs), galactocerebroside, Oligo2 (premyelinating OLs) and, finally, mature myelin antigens such as MBP and PLP (proteolipid protein). The purple and yellow dashed lines indicate the diverse differentiation potential of OPCs, although this signaling pathway is still under debate.

In summary, OPCs show diverse potential for proliferation and differentiation characteristics (Fig. 1). In the presence of CNS injury, OPCs in different regions could generate different cell types in response to noxious stimuli [52]. Thus, fully understanding the origin and development of OL lineage cells is helpful for seeking new therapeutic approaches for the treatment of various neurological and mental diseases.

## White matter integrity in depression

In the CNS, OPCs mainly differentiate into OLs, giving rise to myelin sheaths that enwrap sections of axons, termed internodal regions of nerve fibers; in this manner, they form a special structure called white matter (WM), which is a major component of the CNS. In contrast to gray matter (GM), which consists mainly of neuronal cell bodies, dendrites and conventional synaptic structures for local information processing, WM pathways are part of anatomically specific neural structures that mediate unique capabilities and/or behaviors. Using homotopic- and heterotopic transplantations into the adult mouse cerebral cortex, it was revealed that OPCs from WM differentiate more efficiently into mature, myelinated OLs than GM-derived OPCs [53]. Studies have shown that OPCs generate OLs faster in WM than in GM and faster in younger mice than in older mice [54]. In the human forebrain, the WM is more than half the volume, a 3- to 4-fold increase over the relative WM volume in rodents [55–57]. Complex behavioral dysfunctions such as depression could initially arise when neural traffic in certain WM tracts is altered.

By using magnetic resonance imaging, research studies have shown that decreased WM hyperintensities (WMHs) and abnormalities in myelin integrity start to alter prefrontal regions in the early stages of depression and continue to worsen throughout disease progression without overt changes in OPCs in the WM [58]. For example, WM tract regions in the corpus callosum (CC), the cingulum bundle, the uncinate fasciculus, the ventral prefrontal cortex, the left dorsolateral prefrontal cortex (dlPFC), the anterior limb of the internal capsule, the inferior parietal portion of the left superior longitudinal fasciculus, the temporal cortex, the bilateral corticospinal tracts and other cortical or subcortical areas are decreased significantly [59–68]. Samples from patients diagnosed with depression have revealed reductions in myelin content, axon numbers, and MBP expression as well as the occurrence of reactive gliosis in various brain areas [69, 70].

Regarding structural changes in the depressed human brain, several lines of evidence from animal studies have indicated that WM abnormalities may play a vital role in the pathophysiological mechanisms underlying depression. In a chronic unpredictable stress (CUS) model of depression in rats, Gao et al. found that the WM volume, the total length of myelinated fibers and the mean diameter of myelin sheaths in the WM of the CUS rats were significantly decreased compared to the control group [71]. By using an unpredictable chronic mild stress (UCMS) mouse model, Wang et al. found a significant reduction in myelin and oligodendrocyte-related proteins and observed that stress induced depression-like behaviors and WM deficits in UCMS mice. After treatment with the antidepressant desvenlafaxine, the stress-induced injury of WM OLs and the depression-like phenotypes of UCMS mice were ameliorated via an increase in the phosphorylation of the rate-limiting enzyme of cholesterol synthesis, indicating a possible cellular mechanism of WM injury in depression [72].

Impaired WM integrity is more often seen in association with depressive symptoms in other neurological and psychiatric disorders, including cerebral small vessel disease (SVD) [73, 74], multiple sclerosis [75, 76], Parkinson’s disease [77, 78], schizophrenia [79, 80], bipolar disorder [81, 82], and autism spectrum disorder [83, 84]. For instance, Brooker et al. used path analyses in cross-sectional data to model the relationships among depression, disability, and disability on quality of life (QoL) in SVD stroke and non-SVD stroke patients. An analysis of diffusion tensor imaging data demonstrated a direct correlation between WM damage and depression in the SVD stroke population, which could be due to the disruption of small perforating end arteries supplying the WM [73]. At this time, of course, it is not possible to conclude that the depressive symptoms in these different conditions are mediated by dysfunction of OL lineage cells. It is also possible that another pathological change, unrelated to OL lineage cells, is responsible for depression and that any changes in OL lineage cells are simply coincidental and not causative. That said, a growing body of circumstantial evidence links changes in OL lineage cells to depressive symptoms. Therefore, the disruption of OL lineage cell proliferation and differentiation in the WM may contribute to the depression phenotype.

## Myelin-regulated genes and myelination in depression

Emerging evidence has shown that oligodendrogenesis and myelination in the prefrontal cortex (PFC) are highly sensitive to stressful experiences, including physiological and pathological conditions [85, 86]. The PFC is a critical brain region involved in complex emotional and cognitive behaviors. Liu et al. reported that protracted social isolation decreases myelin gene products and nuclear heterochromatin formation, thus inducing transcriptional and ultrastructural changes in OLs of the PFC, which ultimately leads to impaired adult myelin formation [85]. Indeed, in stress-induced depressive states in mice, 69% of the most significantly downregulated genes were myelin-related such as myelin oligodendrocyte glycoprotein (Mog) and ermin (Ermn) [87]. When preventing adult myelination by conditional deletion of myelin regulatory factor (MRF) in Pdgfra-CreER^T2^; Myrf ^flox/flox^ mice, motor learning and cognitive memory are impaired by the loss of newly formed OLs even in adulthood [88]. Moreover, venlafaxine, a serotonin and norepinephrine reuptake inhibitor antidepressant, can successfully improve cognitive impairment and depression-like behaviors in a cuprizone-induced demyelinated mouse model [89].

The quaking gene (*qki*) encodes a highly conserved RNA binding protein (QKI) that also plays a critical role in the postnatal CNS as a myelination regulator [90, 91]. Although immunohistochemistry shows strong QKI protein expression abundantly but not exclusively in OLs, homozygous mutant quaking viable (qk^v^) mice exhibit a lack of QKI expression in OLs, producing severe dysmyelination that causes quaking movements [92]. Further study also revealed an increased colocalization of the QKI-6 isoform interacting with argonaute 2 (Ago2) and MBP mRNA in cytoplasmic granules of OLs during cellular stress [93]. It is generally accepted that MDD is a disabling disorder and that suicide is its most serious consequence [94]. Over the last decades, there has been a growing interest in the investigation of neurobiological processes involved in major depression and their relationship to suicide [95]. In human species, the messenger RNA (mRNA) levels of multiple transcripts of QKI evaluated on Affymetrix microarrays revealed significant reductions in 11 cortical regions and the hippocampus and amygdala of suicide victims compared with control subjects. The qPCR results were confirmed, as reduced expression of QKI protein was observed in the orbitofrontal cortex (OFC) as well, suggesting a specific role of QKI in myelination-related deficits in the etiology of psychiatric disorders and essential for the determination of myelination OLs in MDD suicide victims [96].

With the sequence of the human genome being publicly available since 2001, gene expression analysis at the transcriptional level is becoming a powerful research tool to study the pathophysiologic underpinnings of human disorders including MDD [97]. Recent microarray analysis of postmortem tissue from depressive individuals revealed that myelination or OL-lineage-related transcriptional genes [98], especially genes that are important for myelin structure (*CNP, MAG, MAL, MOG, MOBP, PMP22, PLLP, PLP1*), were significantly downregulated when the symptoms worsened [60, 98]. In agreement with these observations, animal studies on depression have also demonstrated that values of ~30–40 genes were altered when mice were exposed to chronic unpredictable mild stress. Among them are OL-associated genes such as *MBP*, *MOB* and *CNP* [99–101]. For instance, the *CNP* gene encodes the enzyme 2′,3′‐cyclic nucleotide 3′‐phosphodiesterase (CNP), which is expressed in the development of OLs and increases with the onset of myelination and remains detectable throughout life [102]. A robust reduction in CNP expression at both the mRNA and protein levels has been observed in the postmortem brains of patients with schizophrenia, bipolar disorder or MDD [103, 104]. Although the findings showed that CNP reduction might be critical in a more general disease process and is not restricted to a single diagnostic category for severe mental disorders, in vitro and in vivo evidence demonstrated that aged CNP+/− mice with a 50% reduction in CNP expression exhibit features of depression and catatonia. Further phenotype-based genetic association studies in schizophrenia patients of the Göttingen Research Association for Schizophrenia data collection suggested that the CNP SNP rs2070106 AA genotype influences myelin/axon integrity in the frontal CC fibers, indicating that age- and genotype-dependent association of CNP rs2070106 might be causative of a catatonia-depression syndrome with age [105].

MDD usually affects women approximately twice as often as men. More recently, a large-scale gene expression meta-analysis across corticolimbic brain regions compared male and female MDD patients. The literature revealed multiple transcriptional changes in opposite directions between men and women with MDD [106]. For instance, genes specifically expressed in OLs, such as *MOBP* and *MAG*, were significantly decreased in the anterior cingulate cortex (ACC) and dlPFC but increased in the amygdala (AMY) of women with MDD. However, the opposite gene expression changes occurred in men with MDD, which suggests that MDD has a complex molecular mechanism that may differ between sexes and brain regions [107]. In addition, the latest single-nucleus transcriptomics study from the PFC of MDD cases reported that the changes in gene expression occur predominantly in two cell types: OPCs and deep-layer excitatory neurons. In particular, genes such as *PRNP* (downregulated) and *KAZN* (upregulated) were strongly altered in the OPC cluster, indicating that these genes associated with myelination and synaptic plasticity play a role in the pathogenesis of depression [108].

## Neurotransmitters and their receptors in depression

Among the large array of neurotransmitter receptors present in OL lineage cells, glutamate receptors seem to occupy a key position since they are highly diverse and differentially expressed by progenitors and mature OLs. During brain development, glutamate and GABA signaling play an important role in activity-driven adaptive myelination and thus instruct OPCs to differentiate into myelinated OLs through multiple interactions, including synaptic and/or extrasynaptic modes [109]. OPCs establish physical contacts with functionally relevant neuronal domains and form in parallel with neuronal synaptogenesis, including dendrites, somata, nodes of Ranvier and synaptic cleft [110–112]. It has been shown that elevated glutamate levels and gene expression of glutamate receptors are found in the plasma, cerebrospinal fluid (CSF) and locus coeruleus of postmortem brains from patients with depression [113]. Although the pathological mechanism is unclear, researchers found that MDD patients treated with the antidepressant venlafaxine demonstrated decreased glutamate levels in CSF, indicating a correlation between the attenuation or dysfunction of the glutamatergic system and antidepressant treatment of MDD [114]. Interestingly, a recent study reported that NG2 cleavage by the α-secretase ADAM10 in OPCs impairs N-methyl-D-aspartate (NMDA) receptor-dependent long-term potentiation (LTP) in pyramidal neurons of the somatosensory cortex [115]. Moreover, genetic ablation of NG2 glia with diphtheria toxin in the PFC of the adult brain alters AMPA receptor membrane trafficking and impairs excitatory glutamatergic neurotransmission and extracellular glutamate uptake, which ultimately results in depressive-like behaviors in mice [116]. As NMDA receptor subunit NR1 is mainly expressed in OL processes and AMPA/kainate receptor subunits are mainly found in the somata, any alterations of these receptors could cause glutamate receptor-induced excitotoxicity and glutamate overload in OLs, which could be at least a partial explanation of elevated glutamate levels in depression [117–119].

It is worth mentioning that altered cortical gamma-amino butyric acid neuronal activity and excitatory drive onto the GABAergic interneuron subclass occurred in subjects with schizophrenia [120]. OPCs in the CA1 area of the hippocampus have been shown to receive direct glutamatergic and GABAergic synaptic inputs from neurons [110, 111]. These neuronal–glial excitatory synapses are capable of eliciting a form of LTP that regulates the proliferation and differentiation of pre-OLs. On the other hand, OPC activation-derived GABA release tunes GABAergic synapses and impacts hippocampal excitatory–inhibitory balance, which ultimately causes a psychiatric disorder in a social defeat stress mouse model (Unpublished). Therefore, the interactions between neuronal-OPC synapses might be good candidate targets for potential therapeutic interventions.

Another important endogenous signaling molecule is adenosine, which can both activate P_1_ and P_2_ purinergic receptors. P_1_ adenosine receptors are classical 7-transmembrane-domain metabotropic receptors and can be classified into four types: A_1_, A_2a_, A_2b_ and A_3_ [121]. All the four types of P_1_ adenosine receptors were identified in OL lineage cells at the mRNA level. Activation of P_1_ adenosine receptors inhibits proliferation, stimulates differentiation and promotes myelin formation of OPCs [122]. A study found that macrophage-derived soluble factor such as interleukin-1β causes significant oligodendrocyte cytotoxicity and demyelination in a genetic A1AR null (A1AR^-/-^) mouse. After the treatment with caffeine in a EAE mouse model, it reduces the severity of demyelination by increasing A1AR expressions [123]. Moreover, higher depressive-like behaviors also occurred in A_2a_ receptor-deficient mice as well [124]. P_2_ purinergic receptors contain the ionotropic ATP receptor P_2_X family, including P_2_X_1_ to P_2_X_7_ [125, 126], and metabotropic P_2_Y purinoceptors, which can be broadly divided into P_2_Y_1,2,4,6,11_ and P_2_Y_12,13,14_ groups [127]. OPCs most likely express P_2_X_1,2,3,4,7_ protein in purified postnatal cultures [128], while mature OLs mainly express the P_2_X_7_ subtype [129]. The P_2_X_7_ receptor has been found to be a major factor of the P_2_ receptor family in the pathology of depression, which regulates OL lineage cell proliferation, migration and differentiation, although the role of metabotropic P_2_Y purinoceptors in depression is poorly understood [130]. Evidence of P_2_X_7_ receptors mediating the pathology of depression can be observed as follows. First, P_2_X_7_ receptors in OL lineage cells control the release and uptake of neurotransmitters such as 5-HT, glutamate, GABA, NA and NO under stress and depression conditions [126]. Second, excessive activation of P_2_X_7_ receptors leads to demyelination and cell death of OLs mediated by intracellular Ca^2+^ [131]. Third, genetic deletion of P_2_X_7_ receptors significantly increases basal brain-derived neurotropic factor (BDNF) production in the CNS and results in an antidepressant phenotype in mice [132].

In humans, serotoninergic signals regulate OL development and myelination, and a typical clinical feature of depression is accompanied by an increase in 5-HT in the brain [133]. It has been reported that there are two important 5-HT receptor subtypes expressed in OL lineage cells: 5-HT_1A_ and 5-HT_2A_. Exposure to 5-HT suppressed myelin-related transcriptional factors and therefore resulted in the injury of OL lineage cells and myelination malformation through 5-HT_2A_ receptors [134]. Moreover, excessive stress could activate the HPA axis and increase the level of glucocorticoids that can affect emotion and memory by binding to glucocorticoid receptors (GRs) or mineralocorticoid receptors (MRs), which have been demonstrated to be expressed in different lineages of OLs in various brain regions of adult mice [135]. Hence, the increased 5-HT and/or glucocorticoids might play a role in the inhibition of the proliferation and myelination of OL lineage cells in depressed mammalian brains, although the detailed mechanisms of these two pathways are still under elucidation [136].

It is well known that aberrant dopaminergic transmission in brain neurons dominates the causation of mental diseases such as schizophrenia (SZ), bipolar disorder (BD) and MDD, especially regarding the therapeutic targets for psychosis [137, 138]. However, it is important to point out that alterations of extracellular dopamine (DA) targeting DA receptors affect not only neurons but also OLs. Two DA receptors, D2 and D3, are predominantly expressed in mature OLs and OPCs during myelin formation and play an essential role in myelin maintenance [139, 140]. It has been reported that DA D2 receptor knockout (D2R^-/-^) mice display increased anxiety and depression-like behaviors and a decrease in myelin levels as well upon chronic stress, which possibly inactivates the Wnt/β-catenin signaling pathway in association with DA signaling through D2R in OLs [141]. Hence, considering the malfunction of DA action on DA receptors expressed in OL lineage cells, it is reasonable to hypothesize that OL/myelin defects and neuronal dopaminergic dysfunction form a circuit for etiopathogenesis in depression disorder [142].

## Functions of microRNAs in depression

MicroRNAs (miRNAs or miRs) are a class of endogenous small noncoding RNAs that consist of ~22 nucleotides that have evolved into eukaryotes to regulate a multitude of biological processes by directly binding to their mRNAs through suppressing unwanted genetic materials and transcripts [143]. MicroRNAs were identified in the early 1990s [143], and research continues today. They have been implicated in developmental and neuroplasticity-related processes, such as neurogenesis, differentiation, apoptosis and LTP, in the CNS by controlling multiple aspects of cellular development and homeostasis, including cell fate determination and differentiation [144, 145], and some of them also play a critical role in the development of OL lineage cells [146]. For instance, upon the withdrawal of mitogens, miR-219, one of the most highly OL-specific miRNAs, serves to link the initiation of OL gene expression with the rapid inhibition of OPC proliferation, promoting rapid and coordinated OL differentiation and subsequent myelin formation [147]. Moreover, miR-219 could also play a critical role in promoting myelin repair in the CNS by cooperating with miR-338 [148]. A recent study showed that miR-212 could directly target PLP 1 to regulate the development of OLs [149].

In addition to the aforementioned physiological functions, microRNAs expressed in OL lineage cells also play an important role in mental disorders such as depression. miR-137 is involved in modulating neurogenesis in adult neuronal stem cells and is closely related to cognitive deficits and mental illnesses such as schizophrenia and depression. Repression of miR-137 in the forebrain was associated with increased cognitive performance in a conditional Dicer knockout mouse model [150]. A genome-wide association study recently demonstrated an important role of the miR-137 rs1625579 variant in determining heterogeneity among some patients with mental disorders such as schizophrenia, as well as the onset of MDD and the structural features of the brain [151]. MiR-137 could specifically regulate the *TCF4* gene, which promotes the initial stage of OL differentiation to affect the WM integrity in these mental disorders [151, 152]. Moreover, another study in postmortem brains revealed that miR-21 was largely reduced in the WM and OFC of human subjects with MDD. The colocalization of miR-21 with the OPC-specific markers PDGFRα and MBP by double immunostaining indicated the highly selective expression of miR-21 in OL lineage cell bodies of WM. Further mechanistic studies showed that miR-21 was largely correlated with the regulator STAT3 and myelin-related mRNA to directly link with depression [153].

In addition to the microRNAs expressed by OL lineage cells, which play an important role in depression, exogenous microRNAs could also affect OL lineage cells in the depressed brain. For instance, miR-92a-3p may be associated with WMHs in poststroke depression (PSD). As a common complication of stroke, PSD is characterized by long-lasting, persistent low mood [154]. Patients with WM lesions or elevated miR-92a-3p at baseline were more likely to develop depression within 2 weeks after stroke, and WM impairment was possibly one of the mechanisms of miR-92a-3p in the pathology of PSD [155]. Overall, the functionality of microRNAs in OL lineage cells suggests that they are not only essential during OL development but also actively involved in depression progression and therefore could possibly be used as diagnostic and prognostic markers for depression illness.

## Roles of regulatory factors in depression

Neuregulin 1 (NRG1) is a critical growth factor during brain development, and the *NRG1* gene has been identified as a potential susceptibility gene for some mental disorders, such as schizophrenia or BD [156]. Recent studies have found that the NRG1-ErbB signaling pathway controls the development of OL lineage cells and myelination as well [157]. Blockade of the NRG1-ErbB signaling pathway in OLs not only leads to changes in OL morphology and cell number but also causes mice to exhibit impaired movement and increased depressive-like behaviors, with symptoms resembling those found in patients with depression, schizophrenia, BD and other psychiatric diseases [158–160].

Fibroblast growth factor 2 (FGF2) is a member of the FGF family, which consists of 22 members; there are five known FGF receptors in humans [161]. FGF2 is involved in various biological processes and diseases, such as cell proliferation and differentiation, adult neurogenesis, tumor-induced angiogenesis, cardioprotection, and fracture repair [162–165]. A recent study found that FGF2-FGFR signaling is sufficient and essential for depressive-like behaviors and highlights that the PFC is a main brain region sensitive to antidepressant actions [166]. Focal ablation of OPCs in the PFC impaired glutamate signaling and uptake, which directly induced depressive-like behavioral deficits. Further investigation revealed that knocking down FGF2 expression in prefrontal cortical OPCs recapitulated the anxiety-like phenotype and thus suggested that genetic and stress-induced loss of OPCs triggered the emergence of depressive-like behaviors by reducing the secretion of FGF2 [116].

γ-Secretase, a membrane-embedded aspartate protease, catalyzes peptide bond hydrolysis of a large variety of type I integral membrane proteins exemplified by amyloid precursor protein (APP) [167]. The best-known function of γ-secretase is the cleavage of APP in AD. Nevertheless, numerous studies have found that γ-secretase secreted by OLs controls the development of myelination through activation of Notch1 signaling and plays important roles in neurological and psychiatric diseases such as epilepsy, schizophrenia, autism, obsessive-compulsive disorder and depression [168–171]. It has been reported that altered expression of the γ-secretase complex, especially the largest decrease in nicastrin protein, has been found in reserpine-induced MDD mice, which is correlated with phosphorylated MKP-1, a key factor in the pathophysiology of MDD [172]. However, whether γ-secretase exhibits a causative or correlative function in depression still needs to be carefully identified.

## Inflammatory modulation in depression

The earliest evidence of a relationship between inflammation and depression was found ~30 years ago. When clinicians used recombinant human cytokines interleukin-2 (IL-2) and interferon-α (IFN-α) to boost the immune system to eliminate tumors, they found significant depressive symptoms in patients after therapy [173, 174]. Emerging evidence has demonstrated that inflammation and depression are inseparable. The innate immune system can be activated when people suffer from social stress [175]. Current studies found that stress activates the immune system mainly in two ways, including the HPA axis and the sympathetic nervous system, both of which have immunomodulatory functions [176]. In addition, postmortem brain studies have also demonstrated that the expression of inflammatory cytokines, such as IFN-γ, IL-1α, IL-2, IL-3, IL-5, IL-8, IL-9, IL-12A, IL-13, IL-15 and TNF, is significantly changed in patients with depression and/or associated with the subsequent development of depression [177]. Moreover, some studies even suggest that the increased expression of inflammation markers, including C-reactive protein, acute phase protein, soluble adhesion molecules, tumor necrosis factor, interleukin-1β (IL-1β), IL-6 and/or proinflammatory cytokines, in depressive individuals has become a distinctive clinical indicator of depression [175, 176, 178]. Using anti-inflammatory drugs such as nonsteroidal anti-inflammatory drugs and cytokine inhibitors to treat depression, clinical trials and meta-analyses have proven their antidepressant effects, although the underlying mechanism remains to be clarified [179].

Previous studies mostly focused on the functions of microglia or astrocytes in inflammation during depression due to their quick response to pathogens or tissue damage [10, 180–182]. Mature OLs, however, did not attract attention until very recently; they were revealed as neuroinflammation-related glia, as they were found to express various immunomodulatory molecules, including antigen presenting molecules (MHC class I and II), cytokines and chemokines (IL-1β, IL-18, Il17A, CCL2, CXCL10), complement regulatory molecules (CD46, CD55, CD59) and neuroimmune regulatory proteins (CD200, CD47) [183]. There is not enough evidence to show that OLs have a direct effect on the inflammatory pathology of depression, yet studies have demonstrated that OL lineage cells indeed have indirect modulation in inflammation-induced mood disorders through multiple signaling pathways. As mentioned before, mood disorders such as MDD may have parallels with the psychopathological features observed in multiple sclerosis (MS). Recent clinical reports and epidemiological observations have shown that MDD is indeed associated with MS, which is characterized as a neuroinflammatory disorder with excessive loss of myelin in axons [184–186]. Interestingly, it was found that an increasing number of OPCs and remyelination are usually observed in focal areas infiltrated with activated T cells in MDD patients [187–189], indicating that high expression of CCL-2, IL-33, IL-1β and MMP9 in OPCs possibly participates in the modulation of immunoactivity and is involved in CNS repair processes [190–192]. Another study found that OLs are capable of modulating immune function by producing IL-1β under chronic stress to open up the blood–brain barrier and recruit peripheral immune cells into the brain parenchyma to redirect themselves to areas of demyelination [190, 193, 194]. Moreover, injecting endotoxin lipopolysaccharide (LPS) into the brain could induce WM injury (WMI), OL loss and depression-like behavior in rodents [191, 195]. In vitro studies found that LPS is able to damage OLs when cocultured with microglia by activating innate immunity through the Toll-like receptor 4 (TLR4)-dependent signaling pathway [196, 197]. Furthermore, OL-expressed IL-1 receptors could also participate in the progression of depression. Previous studies revealed the activation of brain immune cells and microglia in people with MDD [198, 199]. Activated microglia are a major source of proinflammatory cytokines, including IL-1, IL-6, and IL-12 release in depression [176, 194]. The activation of microglia-derived IL-1β acts on IL-1 receptors in OL lineage cells, ultimately leading to synaptic deficits and myelin loss possibly through the inhibition of phosphorylation of FYN/MEK/ERK [200]. Taken together, the evidence suggests that immunoactivity modulation of OL lineage cells plays an important role in the cellular pathology of depression and the interaction between inflammation and the integrity of myelinated axons remains to be further investigated in MDD.

## Concluding remarks

**Fig. 2:**
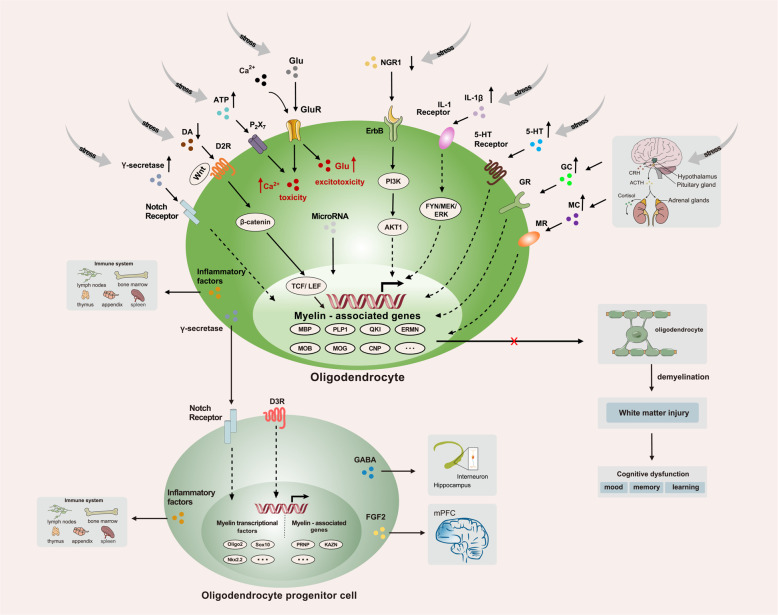
An overview of the functional roles of oligodendrocyte lineage cells in depression. Under extreme social pressure, glutamate, ATP, 5-HT or the adrenal cortical hormone released by the activated hypothalamic–pituitary–adrenal axis stimulates different signaling pathways in oligodendrocyte lineage cells. These signaling pathways may affect myelin gene transcription, myelin-associated genes to cause demyelination or increase [Ca^2+^]_i_ to a toxic level. Moreover, some molecular factors released by oligodendrocyte lineage cells, such as microRNAs, Neuregulin 1, fibroblast growth factor 2 (FGF2), γ-secretase or GABA, also influence the homeostasis of the central nervous system. Under the stimulation of stress, inflammatory factors released by oligodendrocyte lineage cells can directly affect the immune system and cause depression.

Depression is a severe illness that seriously affects human mental health and diminishes QoL worldwide. However, the pathogenesis and pathophysiology of depression are still not fully understood. In the absence of a more complete etiological understanding, it is not surprising that current depression therapies fail to produce lasting benefits in about half of depressed patients. New treatment strategies are urgently needed. Research on glial cells indicates that these cells participate importantly in complex behaviors. OL lineage cells were once thought to have no other function than insulating CNS axons to increase the transmission speed of action potentials. Herein, we have reviewed more recent discoveries indicating that cells of this lineage are vastly more functionally complex than initially thought. They are crucial for certain types of neuroplasticity, express neurotransmitter receptors that make them able to communicate with neighboring neurons and axons, and provide trophic and metabolic support to axons. There is strong circumstantial evidence that OLs are consistently altered in patients with depression and other psychiatric conditions. Moreover, there is unequivocal experimental evidence that genetic alterations in these cells alone can result in major behavioral changes. We conclude that, by the strong suggestion of existing evidence, OL lineage cells are crucial participants in complex human psychological functions such as emotion, memory and cognition. The corollary conclusion is that these cells are likely to contribute to human behavioral alterations such as depression (Fig. 2). The evidence warrants further investigation into the roles of OL lineage cells in the pathogenesis of depression, with the ultimate goal of revealing new and possibly more effective therapies for this debilitating illness.
